# CRISPR screening redefines therapeutic target identification and drug discovery with precision and scalability

**DOI:** 10.1016/j.jpha.2025.101357

**Published:** 2025-06-02

**Authors:** Yao He, Xiao Tu, Yuxin Xue, Yuxuan Chen, Bengui Ye, Xiaojie Li, Dapeng Li, Zhihui Zhong, Qixing Zhong

**Affiliations:** a Laboratory of Neurological Disease Modeling and Translational Research, West China Hospital, Sichuan University, Chengdu, 610041, China; b Medical Innovation Center, Sichuan University of Science & Engineering, Zigong, Sichuan, 643002, China; c Key Laboratory of Drug Targeting and Drug Delivery System of the Education Ministry and Sichuan Province, Sichuan Engineering Laboratory for Plant-Sourced Drug and Sichuan Research Center for Drug Precision Industrial Technology, West China School of Pharmacy, Sichuan University, Chengdu, 610041, China; d Medical College of Tibet University, Lasa, 850002, China; e Tianfu Jincheng Laboratory, City of Future Medicine, Chengdu, 641400, China

**Keywords:** CRISPR-Cas, Gene editing, Targets identification, Drug discovery

## Abstract

Clustered regularly interspaced short palindromic repeats (CRISPR)-Cas9 screening technology is redefining the landscape of drug discovery and therapeutic target identification by providing a precise and scalable platform for functional genomics. The development of extensive single-guide RNA (sgRNA) libraries enables high-throughput screening (HTS) that systematically investigates gene-drug interactions across the genome. This powerful approach has found broad applications in identifying drug targets for various diseases, including cancer, infectious diseases, metabolic disorders, and neurodegenerative conditions, playing a crucial role in elucidating drug mechanisms and facilitating drug screening. Despite challenges like off-target effects, data complexity, and ethical or regulatory concerns, ongoing advancements in CRISPR technology and bioinformatics are steadily overcoming these limitations. Additionally, by integrating with organoid models, artificial intelligence (AI), and big data technologies, CRISPR screening expands the scale, intelligence, and automation of drug discovery. This integration boosts data analysis efficiency and offers robust support for uncovering new therapeutic targets and mechanisms. This review outlines the fundamental principles and applications of CRISPR screening technology, delves into specific case studies and technical challenges, and highlights its expanding role in drug discovery and target identification. It also examines the potential for clinical translation and addresses the associated ethical and regulatory considerations.

## Highlights

• CRISPR screening accelerates therapeutic targets identification and drug discovery.
• CRISPR screening has been broadly applied in the study of various diseases.
• CRISPR screening increases insight into the study of drug mechanisms.
• CRISPR with organoid-based screening enables efficient drug target identification.
• CRISPR technology faces off-target effects, data complexity and ethical challenges.

## Graphical abstract

**Figure:**
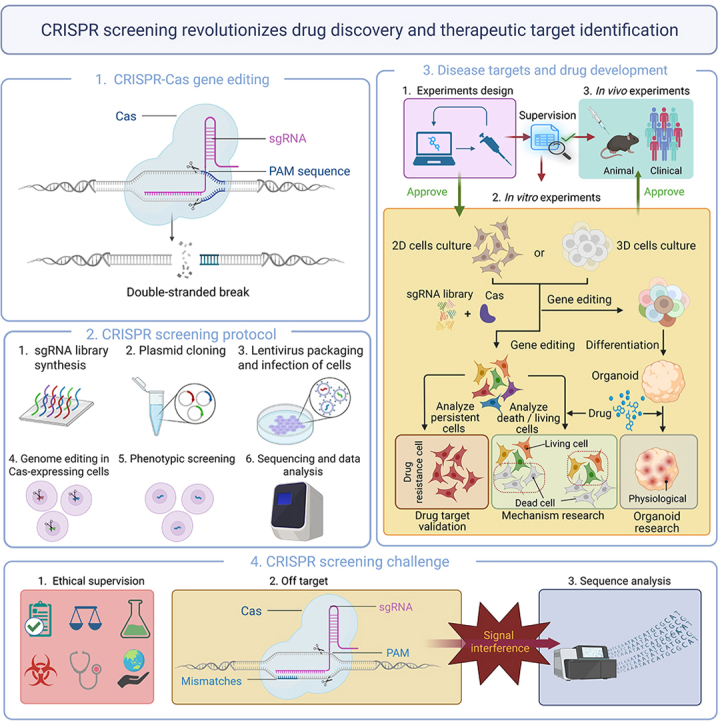

## 1 Introduction

Clustered regularly interspaced short palindromic repeats (CRISPR)-Cas (CRISPR associated protein) technology is a revolutionary gene editing tool, representing the third generation of gene editing methods, following zinc finger nucleases (ZFNs) and transcription activator-like effector nucleases (TALENs) [1,2]. The CRISPR-Cas system, first identified in the 1980s with spacer repeats near the alkaline phosphatase gene in *Escherichia coli* K12 [3], was later recognized as short palindromic repeats by Mojica et al. [4]. Named in 2002 [5], its functional role was confirmed by Barrangou et al. [6] and Marraffini et al. [7]. In 2012, Wiedenheft et al.'s [8] CRISPR-Cas9 discovery revolutionized gene editing. CRISPR-Cas9 employs synthetic RNA guides that specifically bind to the target DNA sequence, leading to Cas9-mediated cleavage and precise genome editing. CRISPR-Cas9, renowned for its simplicity and efficiency, has broad applications in medicine, agriculture, and biological research, enabling the treatment of genetic disorders, and cancer, and advancing gene function exploration [9].

Traditional methods for drug discovery and therapeutic target identification, including proteomics-based techniques [10], such as affinity purification-mass spectroscopy (AP-MS), limited proteolysis mass spectrometry (LiP-MS), and thermal proteome profiling (TPP), structure-based prediction methods (like reverse docking and pharmacophore mapping), and phenotype-based drug discovery (PDD), have significantly advanced our understanding of drug–target interactions and biological mechanisms [11]. Each approach has distinct strengths: proteomics allows high-throughput analysis of drug–protein interactions, structure-based methods rely on three-dimensional (3D) target conformations for *in silico* prediction, and PDD emphasizes physiological relevance by linking compounds to observable phenotypes. However, these methods also face limitations: Proteomics demands rigorous target validation, and structure-based methods are constrained by the availability and accuracy of structural data. PDD often struggles with downstream identification of specific targets. Moreover, traditional cell and animal models may not fully replicate human biology, while single-cell sequencing, despite capturing gene expression patterns, lacks spatial resolution and fails to establish direct gene-function relationships [12]. In contrast, CRISPR screening, based on the CRISPR-Cas gene-editing system, offers a powerful and more direct approach to therapeutic target discovery. It enables genome-wide, high-throughput screening to systematically identify genes associated with specific phenotypes in both *in vitro* and *in vivo* models. This technology perturbs gene function directly, allowing for causal inference between genetic alterations and phenotypic outcomes [13]. Its scalability, precision, and applicability across diverse biological contexts make it a transformative tool in drug discovery, capable of addressing the inherent limitations of conventional approaches.

By designing single guide RNAs (sgRNAs) and constructing sgRNA libraries, CRISPR screening enables targeting a broad spectrum of genes [14]. When combined with specific screening conditions, such as drug treatments or stress-based selection, it facilitates high-throughput cellular screening to identify genes associated with phenotypes. CRISPR screening has proven indispensable in discovering drug targets, enhancing drug effectiveness, and innovating therapeutic approaches [15]. This technique enables the identification of disease-related genes as potential drug targets and allows the evaluation of drug efficacy and safety in specific genetic backgrounds. Furthermore, by editing genes involved in drug metabolism or resistance, it enables the assessment of drug effects on cells under various genetic conditions. Overall, CRISPR screening is a powerful tool in functional genomics and drug discovery, enabling the identification of target genes involved in cell survival, proliferation, differentiation, and specific signaling pathways, thereby advancing disease treatment and drug development [16,17].

Although previous reviews have discussed the mechanisms of the CRISPR-Cas9 system, the high-throughput characteristics of CRISPR screening, whole-genome library construction, and associated technical challenges, they remain largely general in scope and lack a focused analysis on drug-related applications [18,19]. Therefore, in this review, we provide a comprehensive and application-oriented overview of CRISPR-Cas screening, emphasizing its specific roles in drug target identification, elucidation of disease mechanisms, precision drug screening and combined application with organoid research. We further highlight current challenges and future directions, offering novel insights into its translational potential in drug discovery and therapeutic innovation.

## 2 Fundamentals of CRISPR screening technology

### 2.1 CRISPR-Cas system components and mechanism

The CRISPR-Cas system consists of two parts: the CRISPR gene locus and the Cas gene (CRISPR-associated gene). The CRISPR gene sequence is primarily composed of a leader, repetitive sequences (repeat), and spacer sequences (spacer). The leader is located upstream of the CRISPR gene, the transcripts of repetitive sequences can form hairpin structures to stabilize the overall secondary structure of RNA, spacer sequences are foreign DNA sequences captured by bacteria, and when these foreign genetic materials invade again, the CRISPR-Cas system will deliver precise strikes.

**Fig. 1:**
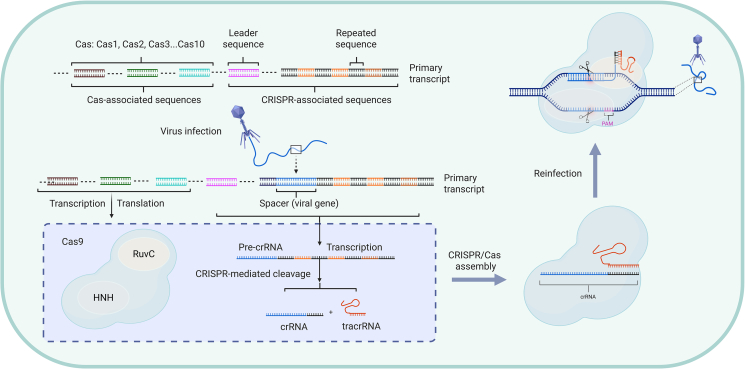
Workflow of the clustered regularly interspaced short palindromic repeats (CRISPR)-Cas9 system. After CRISPR-related sequences are transcribed in cells, the integrated spacer sequences are invaded by viruses. The Cas9 protein binds to CRISPR-derived RNA (crRNA) and trans-activating CRISPR RNA (tracrRNA) to form complexes that recognize and cut foreign DNA. Among them, the RuvC and His-Asn-His (HNH) nuclease domains of Cas9 are responsible for DNA cutting and protecting cells from viruses.

This technology uses the interaction between CRISPR sequence and Cas protein to achieve precise recognition and cutting of specific DNA sequences. As shown in Fig. 1, the CRISPR-Cas system is a complex of Cas protein and the sgRNA. The sgRNA is a fusion of trans-activating CRISPR RNA (tracrRNA) and CRISPR-derived RNA (crRNA) [20]. The complex can specifically recognize the protospacer sequence complementary to the crRNA in the foreign DNA, and the His-Asn-His (HNH) domain of the Cas protein cleaves the DNA strand complementary to the crRNA, while the RuvC endonuclease domain cleaves the additional non-complementary DNA strand [21]. DNA double-strand breaks (DSBs), and expression of foreign DNA is silenced in response to Cas.

In the technical operations of CRISPR-Cas, specific sgRNA design is typically required to recognize the target genome sequence, followed by cloning the sgRNA and Cas coding sequence into appropriate vectors such as plasmids or viruses, and then using methods such as electroporation, liposome-mediated transfection, or virus-mediated transduction to introduce the edited vectors into target cells. Cells will repair the broken DNA through their own DNA repair mechanisms, such as non-homologous end joining (NHEJ) or homologous recombination repair (HDR). Finally, specific screening methods like antibiotics or fluorescence labeling are used to obtain cells with gene editing.

### 2.2 Types of CRISPR screening methods

CRISPR knockout screening can be categorized into two main types: forward screening (gain-of-function screening) and reverse screening (loss-of-function screening). In forward CRISPR screening, the focus lies on analyzing surviving cells to observe which gene knockouts or mutations lead to specific phenotypic changes, such as increased drug resistance or accelerated cell proliferation [22]. This screening method is employed to identify genes that are critical for cell growth, differentiation and survival, or to discover new drug targets [23]. In this approach, a library containing large number of sgRNAs capable of guiding Cas to edit different genes within the cell is constructed and delivered into target cells to create a cellular library, and then subjected to specific selection conditions, under which only cells exhibiting the desired phenotype survive. Finally, the genomic DNA (gDNA) from surviving cells is sequenced and analyzed to confirm successful gene edits, enabling the identification of gene sets associated with the specific phenotype.

In reverse CRISPR screening, cells are sampled at various time points to identify which gene knockouts or mutations lead to increased cell vulnerability to death or other detrimental phenotypes [24]. This screening approach serves to study the role of specific genes in disease onset and progression or to explore combination therapy strategies. Library of sgRNA is introduced into cells, and samples are collected at different times, to identify gene knockouts that affect cell viability or apoptosis. gDNA from these samples is sequenced and analyzed to identify which genes, when knocked out or mutated, lead to unfavorable phenotypic changes.

In addition to CRISPR-Cas knockout screens, several other CRISPR-based approaches have been widely applied in therapeutic target discovery: CRISPR interference (CRISPRi) uses inactive Cas9 (dCas9) fused with transcription inhibitors (such as Krüppel-associated box domain (KRAB)) to bind to the promoter region of target genes, preventing RNA polymerase binding or transcription elongation, thereby inhibiting gene expression. In contrast, CRISPR activation (CRISPRa) combines dCas9 with transcriptional activators to promote gene expression. Using CRISPRa, SRY-box transcription factor 5 (SOX5) was identified as a critical regulator of anti-aging, where its activation led to improved cartilage function in aged mice, demonstrating its relevance in regenerative medicine [25].

Other CRISPR systems have further expanded functional genomic tools. CRISPR-Cas12, like Cas9, edits DNA but with broader protospacer adjacent motif (PAM) recognition, offering greater targeting flexibility. CRISPR-Cas13, on the other hand, targets RNA, making it suitable for studies of transcriptome regulation and RNA virus detection. Additionally, CRISPR droplet sequencing (CROP-seq) combines pooled CRISPR screening with single-cell RNA sequencing, linking genetic perturbations to transcriptomic changes at the single-cell level. This method has been used to investigate T cell receptor signaling in Jurkat cells, enabling the discovery of regulatory networks underlying immune responses [26].

### 2.3 High-throughput screening (HTS) platforms

With the continuous development and improvement of gene editing technologies, high-throughput gene editing CRISPR-based tools and platforms, especially the whole-genome libraries, have become powerful tools for efficient and accurate screening of drugs [27,28]. This technology involves building thousands of libraries of sgRNA and cloning these sgRNA into vectors (e.g. lentiviral vectors) to infect target cells with low multiplicity of infection, enabling simultaneous editing and screening of genes in large quantities. This approach ensures that each cell is infected by one sgRNA, facilitating the subsequent functional screening and identification. Additionally, the gene-modified cells are subjected to specific drug treatments, and the resulting changes in cellular phenotypes are monitored, such as the survival and proliferation rates [29]. The impact of genetic modifications on the cell's response to the drug can then be identified by HTS technology. The CRISPR HTS platform holds revolutionary potential in cancer research, drug development, and gene function analysis by enabling efficient and precise gene editing combined with multi-omics integrative analysis [30].

## 3 CRISPR screening in therapeutic targets identification

### 3.1 Targets discovery and validation

Drug targets identification and validation have been significantly advanced by CRISPR screening, which enables the systematic examination of gene function on a genome-wide scale. By disrupting specific genes and observing phenotypic changes, this method helps pinpoint genes critical to disease processes. Integrating multiple CRISPR screenings from different studies is essential for the discovery of new therapeutic targets. This approach allows researchers to cross-validate findings and identify robust targets across various experimental conditions or cancer types, enhancing the reliability and translatability of the results. For example, Dempster et al. [31] and Pacini et al. [32] integrated CRISPR-Cas9 screening data from the Broad and Sanger Institutes, revealing high consistency across metrics. This integration strengthened cancer-specific analyses, uncovered additional gene dependency biomarkers, and improved the detection of essential genes. Their work provides valuable data support for cancer target discovery and future functional genomics integration.

CRISPR screening has found extensive applications in the studies of various diseases, including cancer, infectious diseases, metabolic diseases, and neurological disorders. In the following sections, we will elaborate on each of these areas respectively.

#### 3.1.1 Targets in cancer

**Fig. 2:**
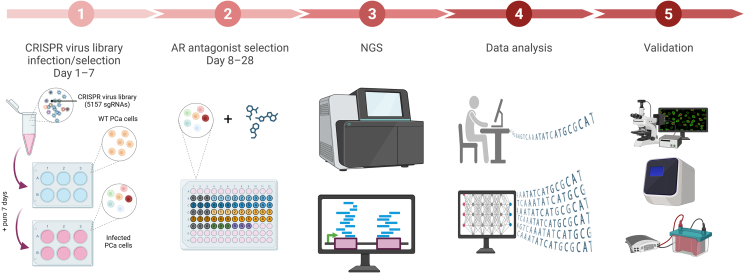
Flowchart for identifying cyclin-dependent kinase 12 (CDK12) as a therapeutic target in prostate cancer using clustered regularly interspaced short palindromic repeats (CRISPR) screening. It involves infecting wild-type prostate cancer cells with a CRISPR virus library, selecting cells treated with an androgen receptor antagonist over 28 days, performing next-generation sequencing (NGS), analyzing the data to identify potential targets, and finally verifying the results through further experimentation.

In recent years, numerous studies have systematically identified key genes essential for the survival of cancer cells and constructed related dependency maps by employing large-scale gene editing screens (such as CRISPR-Cas9) and loss-of-function screens (such as RNA interference (RNAi)), in combination with multi-omics data and protein interaction networks [33,34]. These research efforts not only unveil potential vulnerabilities in cancer but also propose a prioritization framework for anticancer targets based on gene dependency, providing new target resources for personalized cancer therapy [35,36Additionally, the First European Cancer Dependency Map Symposium facilitated exchanges and collaborations among scientists, thereby advancing the development of cancer functional genomics [37]. For instance, Borck et al. [38] analyzed CRISPR screening data from cancer dependency map and identified pelota (PELO) as a novel synthetic lethal target. PELO dependency was observed in cancers with 9p21.3 biallelic deletion and microsatellite instability-high (MSI-H) cancers. In these cancer subtypes, disruption of the super killer complex (SKIc) induces dependency on PELO. Loss of PELO activates the unfolded protein response (UPR), leading to cell death. This finding provides a potential therapeutic target for these cancers and highlights the potential of the cancer dependency map in the discovery of new targets. In addition, Vinceti et al. [39] developed CoRe, an R package for identifying core-fitness genes from CRISPR-Cas9 data. CoRe outperforms existing tools in distinguishing tissue-specific and pan-cancer adaptive genes, aiding cancer target safety assessment and genetic disorder research. In cancer research, by constructing various CRISPR-Cas9 gene knockout libraries and integrating them with HTS methods, researchers can efficiently identify key target genes associated with multiple types of cancers, providing new potential targets and strategies for cancer treatment: CRISPR screening technology has revealed a synthetic lethal relationship of Werner syndrome helicase (WRN) in microsatellite instability (MSI) cancers (such as colorectal cancer, ovarian cancer, and endometrial cancer), providing potential target for the development of novel therapeutic strategies against MSI cancers [35,40,41]. Additionally, Lei et al. [42] introduced a CRISPR-Cas library containing 5157 gRNAs into C4-2 prostate cancer (PCa) cells, after 7-day purinamycin screening, and collected genomic DNA from input, control, and androgen receptor (AR) antagonist groups at days 8–28. Subsequently, sequencing analysis was performed using Illumina NovaSeq 6000, and the sgRNA and genes were ranked by MAGeCK software to generate robust rank aggregation (RRA) scores. Finally, the screening revealed cyclin-dependent kinase 12 (CDK12) as a conserved essential gene for PCa survival, and inhibition of CDK12 using the covalent inhibitor THZ531 demonstrated significant anti-PCa effects (screening process is shown in Fig. 2). Zeng et al. [43] used CRISPR screening to identify methyltransferase-like 1 (METTL1) as a potential therapeutic target for gastric cancer. Wang et al. [44] used a CRISPR library targeting RNA-binding proteins and identified integrator complex subunit 3 (INTS3) as a potential therapeutic target for colorectal cancer (CRC), where INTS3 plays an important role in the progression of CRC by promoting anti-apoptosis and influencing RNA metabolism. Furthermore, the 1,2-dioleoyl-3-trimethylammonium-propane (DOTAP) or cholesterol-MSHINTS3 nanoparticle delivery system was used to inhibit CRC tumor growth. Additionally, Tzelepis et al. [45] conducted large-scale sgRNA library screening and found lysine acetyltransferases 2A (KAT2A) to be the key target for acute myeloid leukemia (AML). The KAT2A inhibitor MB-3 showed promising effects in inducing AML cell differentiation, apoptosis, and growth inhibition. Additionally, Ma et al. [46] leveraged genome-wide CRISPR screening to identify dihydroorotate dehydrogenase (DHODH) as a synthetic lethal vulnerability in multiple endocrine neoplasia 1 (MEN1)-deficient tumors. Mechanistically, MEN1 binds the DHODH promoter to suppress its expression, while MEN1 loss triggers DHODH dependency. Repurposing leflunomide—an approved DHODH inhibitor— demonstrated potent elimination of MEN1-mutant cells in preclinical models, prompting an ongoing clinical trial with promising early results. This work exemplifies CRISPR-driven drug repurposing to accelerate translation of synthetic lethality into precision oncology. Sang et al. [47] performed CRISPR screening in a pancreatic ductal adenocarcinoma (PDAC) model and identified receptor-interacting protein kinase 2 (RIPK2) as a potential target for immunotherapy in pancreatic cancer. In addition, CRISPR screening can rapidly identify genes associated with cancer initiation, progression, and drug resistance, providing crucial targets for new drug development. For example, in hepatocellular carcinoma (HCC) research, CRISPR screening revealed phosphoglycerate dehydrogenase (PHGDH) as a key driver gene of treatment resistance. The use of the PHGDH inhibitor NCT-503 successfully overcame this resistance [48]. CRISPR screening can swiftly validate the efficacy of potential drug targets, thereby accelerating the development of anticancer drugs. In breast cancer research, CRISPR screening identified a synthetic lethal interaction between Haspin kinase and the Aurora-A inhibitor alisertib. Further development of the Haspin inhibitor CHR-6494 significantly enhanced the therapeutic effectiveness of alisertib [49].

Moreover, CRISPR-Cas9 screening, combined with anticancer target prioritization strategies, has been effectively applied to explore the non-coding genome, revealing novel therapeutic targets. Grillone et al. [50] conducted a functional loss-of-function screen on 671 long non-coding RNAs (lncRNAs) in multiple myeloma (MM) cells and their bortezomib-resistant variants. By integrating functional, prognostic, and transcriptional data, they identified 8 oncogenic lncRNAs, with RP11-350G8.5 ranked as the top candidate due to its strong prognostic association. Similarly, Morelli et al. [51] employed CRISPRi to identify MIR17HG and lnc-17-92 as regulators of acetyl-CoA carboxylase (ACACA) via cellular myelocytomatosis oncogene (c-MYC) and WD repeat domain 82 (WDR82) pathways. Optimized antisense oligonucleotides (ASOs) targeting lnc-17-92 demonstrated potent antitumor effects in both *in vitro* and *in vivo* models, completing the target-to-preclinical validation pipeline. Beyond non-coding targets, CRISPR combinatorial screens have identified synthetic lethal interactions that inform precision oncology [52]. For instance, Thompson et al. [53] discovered 27 gene pairs, including FAM50A/FAM50B, exhibiting synthetic lethality (Summarized in Table 1) [38,[42], [43], [44], [45], [46], [47], [48], [49], [50], [51],^53^].

**Table 1:** Applications of clustered regularly interspaced short palindromic repeats (CRISPR) screening in targets discovery.

Type	Disease	Types of CRISPR screening	Targets	Mechanisms	Drug validation	Clinical implications	Refs.
Cancer	Cancers with 9p21.3 biallelic deletion, MSI-H cancers	Forward screening	PELO	Synthesize lethal	/	The potential of the cancer dependence Atlas for the discovery of new therapeutic targets is highlighted.	[38]
	Prostate cancer	Forward screening	CDK12	Immunology	THZ531	Immune checkpoint blocking therapy may work better in prostate cancer with CDK12 mutations.	[42]
	Gastric cancer	Forward screening	METTL1	tRNA modification	/	Modulating the immune microenvironment to influence the effect of immunotherapy.	[43]
	Colorectal cancer	Forward screening	INTS3	RNA processing and stability	DOTAP/cholesterol-mshINTS3 nanoparticle	Potential value of RNA-binding proteins in cancer therapy.	[44]
	AML	Forward screening	KAT2A	Gene transcription regulation	MB-3	It provides new potential targets and therapeutic strategies for AML treatment.	[45]
	Multiple endocrine neoplasia type 1 defect cancer	Forward screening	DHODH	Synthesize lethal	Leflunomide	It is a model of CRISPR-driven new use of old drugs, accelerating the transformation of synthetic lethality into precision oncology.	[46]
	PDAC	Forward screening	RIPK2	Immunology	RIPK2 inhibitor	The discovery of RIPK2 provides a new target for immunotherapy of PDAC.	[47]
	Hepatocellular carcinoma	Forward screening	PHGDH	Drug resistance	NCT-503	By targeting PHGDH, the efficacy of existing drugs can be enhanced to overcome resistance.	[48]
	Breast cancer	Forward screening	Haspin kinase	Synthesize lethal	CHR-6494	The combination of the Haspin inhibitor CR-6494 and the Arora-A inhibitor alisertib is expected to overcome the limitations of single drug therapy and improve the therapeutic effect.	[49]
	Multiple myeloma	Forward screening	RP11-350G8.5, lnc-17-92	Drug resistance, fatty acid synthesis pathway	ASO	It is hoped that new therapeutic strategies can be developed to overcome bortezomib resistance by targeting RP11-350G8.5. The growth and metabolism of tumor cells can be effectively inhibited by targeting lnc-17-92.	[50,51]
	Cancer	Forward screening	FAM50A/FAM50B	Synthesize lethal	/	The importance of homologous genes in synthetic lethality.	[53]
	Serous ovarian cancer (CCNE1-driven cancers)	Forward screening	PKMYT1	Synthesize lethal	RP-6303	RP-6306, as a selective PKMYT1 inhibitor, has shown therapeutic potential in clinical trials in patients with solid tumors with CCNE1 amplification or FBXW7/PPP2R1A inactivation mutations.	[76,77]
	Tumors with high expression of TPST2	Reverse screening	TPST2	IFNγ signaling pathway	/	Inhibition of TPST2 can enhance the effect of anti-PD-1 therapy.	[78]
	Glioblastoma	Forward screening	CARHSP1	Inflammation	/	It is expected to enhance the radiotherapy effect of GBM by inhibiting CARHSP1.	[80]
	Lenvatinib-resistant hepatocellular carcinoma	Forward screening	LAPTM5	Autophagy, drug resistance	Hydroxychloroquine	Lenvatinib resistance can be overcome by knocking out LAPTM5 or utilizing autophagy inhibitors, such as hydroxychloroquine (HCQ).	[81]
Infectious diseases	COVID-19	Forward screening	TMEM106B, PI3K type 3	Endoplasmic reticulum-lysosome pathway	VPS34-IN1, VPS34-IN2, SAR405, and Autophinib	Promoting the development of TMEM106B-targeted drugs. VPS34-IN1 and SAR405, as type 3 inhibitors of PI3K, show broad-spectrum antiviral potential.	[55]
	Dengue virus	Forward screening	SPP	SPP is a key host target of Gideon virus	SPP inhibitor	SPP-targeted compounds showed strong antiviral activity against flaviviruses, which provided promising clues for subsequent optimization.	[57]
	Flavivirus	Reverse screening	TMEM41B	Viral replication	/	By targeting TMEM41B, the replication of multiple flaviviruses and coronaviruses can be simultaneously inhibited, providing new therapeutic strategies to address these global public health threats.	[58]
	H7N9	Forward screening	CYTH2	Viral endocytosis and transport	SecinH3	CYTH2 inhibitors are expected to become a new type of anti-influenza virus drug, providing a new direction for the treatment of influenza virus infection.	[59]
	AIDS	Forward screening	CD4, CCR5, TPST2, SLC35B2, ALCAM	Receptor	/	By targeting these host factors, new anti-HIV drugs can be developed to reduce the virus's ability to enter host cells, thereby lowering the risk of infection.	[61]
	Japanese encephalitis	Forward screening	PRLHR, ASCC3, and ACSL3 etc.	Transcriptional regulation, fatty acid metabolism	/	It provides a new strategy for the treatment of Japanese encephalitis.	[63]
	Herpes virus	Forward screening	CLC-2	Virus replication	Omeprazole	Inhibitors can overcome the resistance of herpes virus and enhance the effectiveness of treatment.	[83]
Metabolic diseases	Diabetes	Forward screening	RNLS, GPR132	Immunology, GPR132-Gi signaling pathway	Pargyline, NOX-6-18	It provides new potential targets and strategies for the treatment of diabetes.	[64,65]
	Atherosclerosis	Forward screening	PCSK9	Regulation of low-density lipoprotein cholesterol	Alirocumab, evolocumab, and inclisiran	Alirocumab and evolocumab have shown good lipid lowering effects and safety in clinical trials.	[66]
	NAFLD	Forward screening	CYP46A1	Modulation of cholesterol hydroxylation	/	The discovery of CYP46A1 provides new molecular mechanism clues for studying diseases related to lipid metabolism abnormalities.	[67]
	Obesity	Forward screening	BSCL2, PLIN1	Influence the synthesis of fat and the formation of lipid droplets	/	It reveals the molecular mechanism of adipocyte differentiation and lipid droplet formation, providing a new perspective on the pathogenesis of metabolic diseases.	[87]
Neurological diseases	AD	Forward screening	Neddylation pathway	A protein modification process similar to ubiquitination	MLN4924	The neddylation pathway plays an important role in maintaining neuronal health.	[69]
	PD	Forward screening	Rab 12, LRRK2	LRRK2 pathway	MLi-2	By targeting Rab12 or its interaction with LRRK2, it is expected to develop more effective PD therapeutic strategies, thereby improving the prognosis of patients.	[71]

MSI-H: microsatellite instability-high; PELO: pelota; CDK12: cyclin-dependent kinase 12; METTL1: methyltransferase-like 1; DOTAP: 1,2-dioleoyl-3-trimethylammonium-propane; INTS3: integrator complex subunit 3; AML: acute myeloid leukemia; KAT2A: Lysine acetyltransferases 2A; DHODH: dihydroorotate dehydrogenase; PDAC: pancreatic ductal adenocarcinoma; RIPK2: receptor-interacting protein kinase 2; ASO: antisense oligonucleotides; PHGDH: phosphoglycerate dehydrogenase; PKMYT1: protein kinase membrane associated tyrosine/threonine 1; CCNE1: cyclin E1; TPST2: Tyrosylprotein Sulfotransferase 2; IFNγ: interferon γ; CARHSP1: calcium-regulated heat stable protein 1; GBM: glioblastoma; LAPTM5: lysosomal protein transmembrane 5; COVID-19: 2019 Coronavirus Disease; TMEM106B: transmembrane protein 106B; SPP: signal peptide peptidase; CYTH2: cytohesin 2; AIDS: acquired immunodeficiency syndrome; HIV: Human immunodeficiency virus; RNLS: renalas; CLC-2: chloride channel 2; GPR132: G Protein-Coupled Receptor 132; PCSK9: proprotein convertase subtilisin/kexin type 9; NAFLD: non-alcoholic fatty liver disease; CYP46A1: cytochrome P450 46A1; BSCL2: Berardinelli-Seip congenital lipodystrophy type 2; PLIN1: perilipin 1; AD: Alzheimer’s disease; PD: Parkinson’s disease; LRRK2: leucine-rich repeat kinase 2; /: no data**.**

In summary, CRISPR screening offers significant advantages in cancer research, including high-throughput and precise gene function studies, discovery of novel drug targets and resistance mechanisms, *in vivo* screening that mimics the tumor microenvironment, multi-dimensional gene regulation capabilities, accelerated drug development and clinical applications, in-depth analysis combined with single-cell sequencing, and diverse screening platforms. These strengths make CRISPR screening an indispensable tool in cancer research, providing robust support for understanding cancer mechanisms and developing new therapies.

#### 3.1.2 Infectious diseases targets

Severe acute respiratory syndrome coronavirus 2 (SARS-CoV-2) is the pathogen responsible for the 2019 coronavirus disease (COVID-19) pandemic [54]. Baggen et al. [55] in 2021 used genome-wide CRISPR screening with the Brunello library to identify key host factors for SARS-CoV-2 infection. Screening in Huh7 cells highlighted transmembrane protein 106B (TMEM106B) and phosphatidylinositol 3-kinase (PI3K) type 3 as essential factors. PI3K type 3 inhibitors (VPS34-IN1, SAR405) blocked SARS-CoV-2 and other coronaviruses, making it a promising broad-spectrum antiviral target, while TMEM106B-targeting drug development remains in early stages. Besides, mosquito-borne flaviviruses, such as dengue virus (DENV), Zika virus (ZIKV), West Nile virus, and yellow fever virus, pose a significant public health threat globally [56]. Qiao et al. [57] constructed a CRISPR-Cas9 library targeting the druggable genome and identified signal peptide peptidase (SPP) as a key host target for Gideon virus. SPP-targeting compounds demonstrated potent antiviral activity against flaviviruses, providing promising leads for further optimization. This study established a platform for identifying host-based drug targets for broad-spectrum antiviral development. Hoffmann et al. [58] performed a genome-wide loss of function CRISPR-Cas9 screen and found that TMEM41B is a flavivirus host factor. Moreover, Yi et al. [59] used genome-wide CRISPR-Cas9 screening in A549 cells to identify cytohesin 2 (CYTH2) as a potential therapeutic target for H7N9 influenza virus infection, confirming its role in viral endocytosis and transport, and validating the antiviral efficacy of CYTH2 antagonists like SecinH3. In addition, Acquired immunodeficiency syndrome (AIDS) is caused by infection with the human immunodeficiency virus (HIV) [60]. Park et al. [61] applied genome-wide CRISPR screening to identify host factors essential for HIV infection. GXRCas9 cells were employed and GFP-negative cells were sorted post-HIV infection, and five key host factors—cluster of differentiation 4 (CD4), C-C motif chemokine receptor 5 (CCR5), tyrosylprotein sulfotransferase 2 (TPST2), solute carrier family 35 member B2 (SLC35B2), and activated leukocyte cell adhesion molecule (ALCAM)—as crucial for HIV infection. Moreover, Japanese encephalitis (JE) is a mosquito-borne zoonotic disease caused by the Japanese encephalitis virus (JEV), posing a serious threat to global public health [62]. Liu et al. [63] used GeCKO library-based CRISPR screening to identify host factors critical for JEV replication. This study revealed key factors like prolactin releasing hormone receptor (PRLHR), activating signal cointegrator complex subunit 3 (ASCC3), and acyl-CoA synthetase long chain family member 3 (ACSL3). Validation included gene knockouts (e.g., EPH receptor A2 (EPHA2), and ACSL3) and siRNA knockdowns (e.g. ASCC3, and PRLHR), which confirmed reduced JEV mRNA levels and viral titers without impacting cell viability (Summarized in Table 1) [55,[57], [58], [59],^61^,^63^].

These mechanisms of virus-host interactions revealed by CRISPR technology are driving a shift in antiviral therapy from a "virus-centric" to a "host-centric" approach. By targeting host dependency factors such as PI3K type 3 and SPP, new avenues for broad-spectrum drug development against SARS-CoV-2, flaviviruses, and others have emerged. Meanwhile, discoveries of genetic polymorphisms (e.g., TMEM41B mutations) are advancing precision medicine. The dual roles of functional molecules like TMEM106B (related to both infection and neurodegenerative diseases) highlight the potential for cross-disease therapeutic targets. Additionally, intervention strategies targeting host factors have not only optimized the control of mosquito-borne viruses but also provided new directions for achieving a functional cure for HIV. These advancements signify a transformation in human antiviral efforts from reactive responses to proactive defense. Despite challenges in clinical translation, they lay the groundwork for constructing virus-host interaction maps and developing multi-target synergistic drugs.

#### 3.1.3 Metabolic diseases targets

Metabolic diseases are a class of disorders caused by disruptions in the body's metabolic processes, involving abnormalities in the metabolism of various substances such as carbohydrates, fats, and proteins. Cai et al. [64] employed the CRISPR-Cas9 system to transduce NIT-1 β-cells with a mouse GeCKO library, and identified renalas (RNLS) as a key target for protecting β-cells. Structural analysis revealed that the US Food and Drug Administration (FDA)-approved drug Pargyline could target RNLS, and oral administration of Pargyline protected transplanted β-cells in diabetic mice, delaying diabetes onset. Similarly, Wang et al. [65] found that oxidized fatty acid metabolites activate G protein-coupled receptor 132 (GPR132), playing a key role in reprogramming islet-resident macrophages and triggering islet inflammation. They developed NOX-6-18, a high-affinity GPR132 antagonist, which significantly reduces islet inflammation and improves glucose metabolism in diabetic mice. Furthermore, proprotein convertase subtilisin/kexin type 9 (PCSK9), a key target discovered through CRISPR gene editing, promotes low-density lipoprotein receptor (LDL-R) degradation, reducing liver LDL uptake. CRISPR-based PCSK9 editing mimics natural loss-of-function mutations, which are associated with lower low-density lipoprotein cholesterol (LDL-C) and reduced cardiovascular risk. Monoclonal antibodies like alirocumab and evolocumab inhibit PCSK9, boosting LDL-R expression to lower LDL-C. Meanwhile, inclisiran, an mRNA-targeting drug, reduces PCSK9 production, cutting LDL-C by over 50% with twice-yearly dosing [66]. Similarly, Si et al. [67] conducted genome-wide CRISPR-Cas9 screening in L02 liver cells treated with oleic and palmitic acids. Genes linked to lipid droplet accumulation, such as cytochrome P450 46A1 (CYP46A1), was identified as a regulator of lipid metabolism. Downregulation of CYP46A1 increased lipid droplet accumulation, while overexpression reduced it, confirming its role in lipid metabolism (Summarized in Table 1) [[64], [65], [66], [67]].

In diabetes research, RNLS was identified as a protector of β-cells and demonstrated new application potential for Pargyline while the GPR132 antagonist NOX-6-18, offered a fresh approach to regulating islet inflammation. In cardiovascular health, PCSK9 inhibitors such as alirocumab and the mRNA-targeting inclisiran effectively lower cholesterol by increasing LDL receptor levels, thereby revolutionizing lipid management strategies. For lipid metabolism, CYP46A1 was shown to be a critical regulator of lipid homeostasis with its modulation directly impacting lipid accumulation thereby paving the way for new treatments for fatty liver disease. These breakthroughs not only expedite drug development across diverse conditions but also propel advancements in precision medicine and targeted therapies.

#### 3.1.4 Neurological diseases targets

Alzheimer’s disease (AD) is a progressive neurodegenerative disease that affects millions of people worldwide [68]. Saurat et al. [69] employed the Brunello CRISPR Knockout library to screen genes during human pluripotent stem cells (hPSCs) differentiation into cortical neurons. Cas9 was induced by doxycycline, and comparisons of wild-type and APPswe/swe mutant cells identified genes depleted in AD's neurons. The neddylation pathway was identified as a regulator of neuronal aging and AD neurodegeneration, with MLN4924 inhibition exacerbating neuronal degeneration. Parkinson’s disease (PD) is the second most common neurological disorder in humans after AD [70]. In addition, Dhekne et al. [71] used genome-wide CRISPR screening with the Brie gRNA library in NIH-3T3 cells to identify factors regulating the leucine-rich repeat kinase 2 (LRRK2) pathway in PD. Changes in phosphorylated Rab10 levels were monitored, identifying Rab12 as a key activator of LRRK2 kinase. MLi-2 was validated as a potential therapeutic agent, inhibiting LRRK2 activity, reducing Rab10 phosphorylation, and improving neuronal damage. These two studies collectively demonstrate the unique advantages of CRISPR technology in unraveling the mechanisms of complex neurodegenerative diseases, paving a new path for developing pathway-specific intervention strategies (Summarized in Table 1) [69,71].

### 3.2 Gene-drug interaction analysis

CRISPR screening, based on chemical genomics, is also a powerful tool for analyzing gene-drug interactions. This approach enables the determination of compound targets, advancing the understanding of drug mechanisms and potential therapeutic applications [72]. Chemical genomics is an interdisciplinary field that integrates chemistry, biology, and informatics to study gene functions and their roles in biological processes using small-molecule compounds [73]. The core principle involves employing specific small molecules as "chemical probes" that modulate the function or signaling pathways of particular proteins within the cells [74]. The CRISPR HTS enables the rapid evaluation of large numbers of compounds to identify those with desired biological effects [75]. Transcriptome analysis, performed by comparing cellular gene expression profiles before and after treatment, reveals regulated gene networks using advanced sequencing techniques. Functional validation experiments, combined with computational modeling, provide deeper insights into the mechanisms of these compounds and assess their potential as therapeutic agents. Overall, chemical genomics serves as a robust platform for drug discovery, target identification, and exploring disease mechanisms.

For instance, Gallo et al. [76] combined CRISPR screening with chemical genomics to identify a synthetic lethal relationship between PKMYT1 and CCNE1 amplification, revealing PKMYT1 as a therapeutic target in CCNE1-driven cancers. Building on this discovery, in the same year, Szychowski et al. [77] developed RP-6306, a first-in-class PKMYT1 inhibitor. These findings underscore the potential of CRISPR-guided drug discovery to rapidly bridge mechanistic insights into clinically actionable therapies, particularly for cancers with genomic vulnerabilities like CCNE1 dysregulation. Oh et al. [78] in 2024 leveraged *in vivo* CRISPR screening to uncover that TPST2 inactivation potentiates anti-programmed cell death 1 (PD-1) therapy by augmenting interferon γ (IFNγ) signaling and antigen presentation via reduced sulfation of IFNγ receptor 1 (IFNGR1). Their findings propose TPST2 inhibition as a combinatorial strategy for high-TPST2 tumors, showcasing CRISPR's capacity to pinpoint actionable targets for precision immunotherapy. These studies highlight how the integration of CRISPR and chemical genomics is uncovering complex disease mechanisms and advancing personalized treatment strategies, signaling a new era of precision and innovation in drug discovery (Summarized in Table 1) [[76], [77], [78]].

### 3.3 Drug resistance mechanisms

Drug resistance mechanisms refer to the ability of parasites, microorganisms (such as bacteria, viruses, mycobacteria), and tumor cells to tolerate chemotherapeutic agents, resulting in diminished or complete loss of drug efficacy. CRISPR screening is also a powerful tool for identifying genes responsible for drug resistance in cancer and infections, offering new strategies for disease treatment [22,79]. For example, Zhu et al. [80] identified calcium-regulated heat stable protein 1 (CARHSP1) as a key driver of radiation resistance in glioblastoma (GBM) through genome-wide CRISPR-Cas9 activation screening. CARHSP1, upregulated in radiation-resistant GBM cells, mediates resistance through inflammatory signaling activation, indicating it as a potential therapeutic target to enhance GBM radiotherapy efficacy. Pan et al. [81] discovered lysosomal protein transmembrane 5 (LAPTM5) as the primary cause of resistance to the targeted drug lenvatinib in hepatocellular carcinoma (HCC) through a genome-wide CRISPR-Cas9 knockout screening. LAPTM5 enhances autophagy and drives lenvatinib resistance in HCC, with autophagy inhibition or LAPTM5 knockout enhancing lenvatinib's anticancer efficacy. Yan et al. [82] used CRISPRi and knockout libraries combined with liquid chromatography-mass spectrometry (LC-MS)-based metabolomics and chemical-genetic interaction analysis to identify resistance genes affecting Delamanid and Pretomanid efficacy against *Mycobacterium tuberculosis (Mtb)*. Genome-wide CRISPR screening revealed rv2073c as a resistance gene and mutations in rv0078 as contributors to increased drug resistance, guiding the development of novel anti-tuberculosis (TB) therapies targeting these pathways. In addition, Yang et al. [83] employed genome-wide CRISPR screening technology in haploid mouse embryonic stem cells to identify chloride channel 2 (CLC-2) as critical gene for herpesvirus replication. The effectiveness of CLC-2 inhibitors, including omeprazole and DIDS, was validated through functional assays and anti-viral testing, demonstrating their ability to overcome herpesvirus drug resistance and enhance treatment efficacy. These studies enhance our understanding of drug resistance and combine gene editing with multi-omics to speed up precision medicine, offering new solutions from target discovery to drug development (Summarized in Table 1) [80,81,83].

In addition, the data integration of pharmacology and CRISPR screening data is changing drug development from a “trial and error” approach to a “mechanical-driven” approach. For instance, Gonçalves et al. [84] linked drug sensitivity data (from 199,219 anticancer drugs) with CRISPR data (from 484 cancer cell lines) and found a positive correlation between drug sensitivity and target knockout. They also found a new link between mitochondrial E3 ubiquitin protein ligase (MARCH5) and myeloid leukemia 1 (MCL1) inhibitor sensitivity, providing new biomarkers and insights into resistance mechanisms. This integration helps overcome drug resistance and improve treatment accuracy.

### 3.4 Target gene network analysis in complex diseases

Gene expression network analysis employs systems biology approaches to integrate and analyze large-scale gene expression data, uncovering the complexity and dynamics of gene regulatory networks. It identifies co-expression patterns of genes, enhancing our understanding of cellular functions and the molecular mechanisms of diseases [85]. For instance, Zhou et al. [86] employed single-cell CRISPR screening with a dual-targeting sgRNA vector to dissect transcription factor networks in CD8^+^ T cells *in vivo*. This approach linked regulators like Bach2, Bcl6, and Foxo1 to distinct cytotoxic T lymphocyte (CTL) programs—including exhaustion, stemness, and effector function—uncovering actionable checkpoints in CTL differentiation. Their work establishes a roadmap for targeting transcriptional drivers to enhance cancer immunotherapies. Jiao et al. [87] employed CRISPR screening to knock out genes associated with metabolic diseases, analyzing morphological changes during adipocyte differentiation and identifying a set of genes that influence lipid accumulation. The direct interaction between Berardinelli-Seip congenital lipodystrophy type 2 (BSCL2) and perilipin 1 (PLIN1) proteins on lipid droplets and the regulation of 1-acylglycerol-3-phosphate O-acyltransferase 2 (AGPAT2) by CCAAT enhancer binding protein alpha (CEBPA) were validated, revealing mechanisms underlying lipid droplet formation, maturation, and adipocyte differentiation, providing insights into the molecular basis of metabolic diseases. These studies collectively demonstrate the translational medical value of gene network analysis in decoding complex disease mechanisms and discovering personalized therapeutic targets, marking a leap in precision medicine from single-target approaches to the era of network regulation (Summarized in Table 1) [87].

## 4 CRISPR screening in drug discovery

### 4.1 Development and screening of candidate drugs

In addition to identifying essential genes, uncovering disease mechanisms, and advancing functional genomics, forward CRISPR screening is also used to discover drug targets [23]. Upon confirming the functionality of potential drug targets identified through CRISPR screening, advanced techniques such as computer-aided drug design (CADD) and HTS are employed to identify candidate compounds that can specifically bind to these targets [88]. For instance, Lampson et al. [89] used CRISPR screening and cryo-electron microscopy to identify STT3 oligosaccharyltransferase (OST) complex catalytic subunit A (STT3A) as a drug target in the nuclear factor kappa-light-chain-enhancer of activated B cells (NF-κB) pathway. An NGI-1 analog was found to inhibit STT3A's catalytic activity, serving as a specific OST-A (the complex containing STT3A) inhibitor and a potential strategy for developing anti-inflammatory drugs. Baumgartner et al. [90] in 2023 identified protein tyrosine phosphatase non-receptor type 2 and 1 gene (PTPN2 and PTPN1) as drug targets via CRISPR screening, revealing that their depletion enhances tumor sensitivity to immune checkpoint blockade. A dual-targeting molecule was designed to bind both phosphatases by optimizing molecular properties, including weight, sp3 hybridization, and clearance rate, leading to the development of ABBV-CLS-484 (AC484). *In vitro* studies confirmed that AC484 inhibits PTPN2 and PTPN1, enhancing Janus kinase-signal transducer and activator of transcription (JAK-STAT) signaling, reducing T-cell dysfunction, and boosting antitumor immune responses. AC484 is the first active-site phosphatase inhibitor to enter clinical evaluation for cancer immunotherapy, potentially paving the way for other therapies targeting this important class of enzymes. Zhao et al. [91] employed CRISPR HTS and chemical genomics to validate BDW568 as a type I interferon (IFN–I) activator. Using a CRISPR platform linking the suicide gene iCasp9 to BDW568's activity, loss-of-function screens identified stimulator of interferon genes (STING), carboxylesterase 1 (CES1), and other genes critical to its mechanism. Kasap et al. [92] developed DrugTargetSeqR, a genomics-based and CRISPR-Cas9-driven drug target analysis method, and used it to identify kinesin-5 as the target of ispinesib. Chu et al. [93] used CRISPR-Cas9 screening in HK-2 cells to identify genes linked to cisplatin-induced acute kidney injury (C-AKI). They found apilimod could alleviate cisplatin's nephrotoxicity. Further research showed that apilimod inhibits phosphoinositide kinase focal vascular endothelial (PIKfyve), promoting transcription factor EB (TFEB) nuclear translocation and enhancing peroxisome proliIerators-activated receptor γ coactivator 1 alpha (PGC1α)-mediated fatty acid oxidation, thereby reducing lipid accumulation and mitigating apoptosis, inflammation, and reactive oxygen species (ROS) production, effectively alleviating C-AKI. These studies all show that CRISPR screening can be used to discover candidate drug targets and elucidate their mechanisms of action, accelerating the entire process from drug development to target validation and marking a seamless transition from laboratory research to clinical applications in personalized medicine (Summarized in Table 2) [[89], [90], [91], [92], [93]].

**Table 2:** Applications of clustered regularly interspaced short palindromic repeats (CRISPR) screening in the development and screening of candidate drugs.

Candidate drugs	Types of CRISPR screening	Disease	Targets	Mechanisms	Clinical implications	Refs.
NGI-1	Forward screening	Inflammation	STT3A	NF-κB signaling pathway	It provides a rationale for developing STT3A-specific inhibitors.	[89]
ABBV-CLS-484	Forward screening	Cancer	PTPN2, PTPN1	JAK-STAT signaling pathway	ABBV-CLS-484 is the first active site phosphatase inhibitor to enter clinical evaluation for cancer immunotherapy.	[90]
BDW568	Forward screening	Cancer	STING, CES1	STING signaling pathway	By targeting the STING pathway, BDW568 is expected to serve as a novel immunomodulator for the treatment of viral infections or cancer.	[91]
Ispinesib	Forward screening	Cancer	Kinesin-5	Mitosis	Developed a genomics-based and CRISPR/Cas9-driven drug target analysis method: DrugTargetSeqR.	[92]
Apilimod	Forward screening	C-AKI	PIKfyve	PIKfyve/TFEB/PGC1α signaling axis	A new drug, apilimod, has been discovered for the treatment of C-AKI.	[93]
Bedaquiline	Reverse screening	Tuberculosis	Rv0678, pepQ	Drug resistance	It provides new targets and strategies for the precision treatment of tuberculosis, which is expected to improve treatment outcomes and reduce the development of drug resistance.	[96]
Linezolid	Forward screening	Tuberculosis	TsnR	Drug resistance	It guides the development and treatment of future tuberculosis drugs	[97]

STT3A: STT3 oligosaccharyltransferase complex catalytic subunit A; NF-κB: nuclear factor kappa-B; JAK-STAT: janus kinase-signal transducer and activator of transcription; PTPN2/1: protein tyrosine phosphatase non-receptor type 2/1 gene; STING: stimulator of interferon genes; CES1: carboxylesterases; C-AKI: cisplatin-induced acute kidney injury; PIKfyve: phosphoinositide kinase focal vascular endothelial; TFEB: transcription factor EB; PGC1α: eroxisome proliferator-activated receptor γ coactivator 1-alpha; TsnR: translation suppressor nucleotide resistance; Rv0678: transcriptional regulator MmpR5; pepQ: Xaa-Pro aminopeptidase.

### 4.2 Integration with phenotypic screening

Integrating CRISPR screening with disease-specific phenotypic targets offers a robust strategy to greatly improve the efficiency of identifying mechanisms of drug resistance and developing novel therapeutic approaches. This method combines the precision of CRISPR gene editing with the detailed insights into biological responses offered by phenotypic screening [94]. It is known that TB remains a significant challenge to global health, highlighting the urgent need for new anti-TB drugs [95]. The drug development involves phenotypic screening to identify active compounds, followed by CRISPR-based editing of TB-relevant genes to create mutants for compound sensitivity testing. This approach uncovers genetic determinants, facilitating the identification of drug targets and biomarkers. For instance, Yan et al. [96] combined CRISPR-KO and CRISPRi libraries to identify essential *Mtb* survival genes (e.g., atpE) and anti-TB drug bedaquiline (BDQ) resistance genes (e.g., Rv0678, and pepQ), as well as BDQ sensitivity genes, including lprG, embA, and topA. Li et al. [97] constructed a CRISPRi library that nearly covers all genes of *Mtb* and conducted chemical-genetic interaction screening using 9 drugs (including seven anti-TB clinical drugs and two non-TB drugs). The study identified a common sensitization target in the mycolic acid-arabinogalactan-peptidoglycan complex for rifampicin, vancomycin, and BDQ, and revealed resistance mechanisms, such as the role of translation suppressor nucleotide resistance (TsnR) in linezolid resistance and elongation translation translation associated (ettA) mutations in multidrug resistance. Additionally, a novel *Mtb* subline with increased sensitivity to macrolides was found, suggesting clarithromycin as a potential treatment. These studies not only accelerate the decoding of drug resistance mechanisms but also drive the leap in target discovery from single genes to functional networks, bringing new opportunities for "repurposing old drugs" and combination therapies in tuberculosis treatment, highlighting the revolutionary value of CRISPR technology in addressing global health challenges (Summarized in Table 2) [96,97].

## 5 CRISPR screening in human organoid studies

**Fig. 3:**
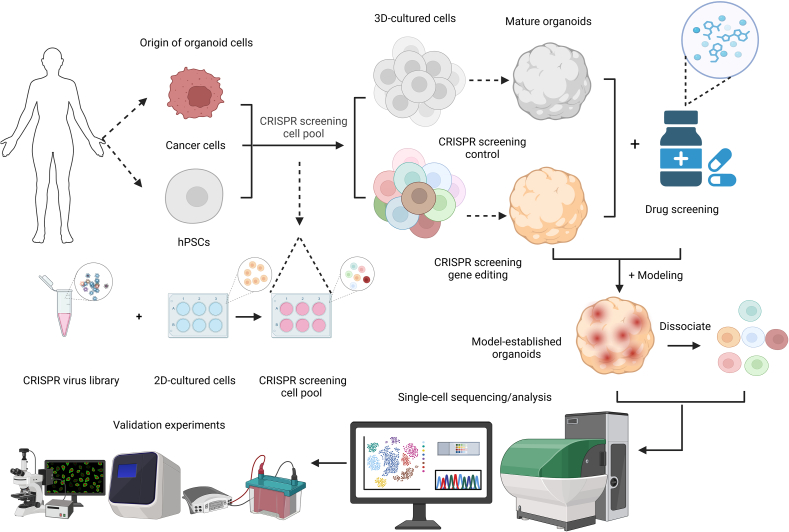
Flowchart of target screening in human organoids using clustered regularly interspaced short palindromic repeats (CRISPR) screen. First, cancer cells and human pluripotent stem cells (hPSCs) were obtained from the human body and two-dimensitonal (2D) cultured cells were infected by the CRISPR virus library. The pool of screened cells was then used for three-dimensional (3D) culture to form organoids and for CRISPR screening control and gene editing. Subsequently, the mature organoids were dissociated into single cells for single-cell sequencing and analysis through drug screening and modeling. Finally, experiments were carried out by microscopy and other equipment to confirm the validity of the screening results.

Human organoids can replicate the complex structure and function of human organs, addressing species differences between humans and animal models, making them an ideal tool for preclinical drug research in human diseases [98]. Since the first organoid (intestinal) was created in 2009, human organoid technology has been widely applied in personalized medicine and drug development [99]. Several drugs that have used this technology for preclinical studies have been approved by the US FDA for clinical trials [100]. This development has created opportunities to use organoid-related research as the initial "phase zero" for clinical trials, improving clinical translation rates. Employing CRISPR technology, pathogenic mutations can be introduced or corrected in organoid models to simulate disease states, evaluate drug sensitivity, and investigate the molecular mechanisms underlying drug action. Patient-derived organoids (PDOs) from human cancer cells, as well as specialized organoids generated from hPSCs, can also be utilized for high-throughput drug screening tailored to individual patients. This approach provides strong support for personalized medicine and accelerates the development of targeted therapies (Fig. 3).

### 5.1 CRISPR screening in patient-derived organoids

There have been many studies using CRISPR screening to find targets using PDO. For instance, Hirt et al. [101] utilized CRISPR-Cas9 technology in human pancreatic cancer PDOs to investigate the impact of AT-rich interaction domain 1A (ARID1A) and breast cancer 2 (BRCA2) gene mutations on drug sensitivity. They discovered that ARID1A missense mutations were associated with increased sensitivity to dasatinib and VE-821. Additionally, through drug HTS, they identified 26 effective drugs from 1172 US FDA-approved compounds, including emetine and ouabain, which kill PDAC cells by disrupting their response to hypoxia. In addition, Jones et al. [102] used CRISPR technology to identify puromycin-sensitive aminopeptidase (NPEPPS) as a key target associated with platinum drug resistance. Through multi-omics analysis, cell experiments, animal models, and bladder cancer PDOs, they validated that the NPEPPS inhibitor tosedostat enhances the sensitivity of bladder cancer cells to cisplatin. This study provides a novel strategy for overcoming platinum resistance. Besides, Madorsky Rowdo et al. [103] utilized CRISPR-Cas9 technology to perform a kinome screen in PDOs from African ancestry breast cancer patients. They identified key kinases such as epidermal growth factor receptor (EGFR), cyclin-dependent kinase 2 (CDK2), and cyclin-dependent kinase 13 (CDK13) and demonstrated that the combination of EGFR and fibroblast growth factor receptor 1 (FGFR1) inhibitors synergistically suppressed the proliferation of breast cancer organoids. HTS study further validated these findings, providing new therapeutic targets and combination strategies for breast cancer treatment. Song et al. [104] identified ATP-binding cassette transporter C1 (ABCC1) as a key gene for MRTX849 resistance in KRAS-G12C mutant non-small cell lung cancer (NSCLC) using CRISPR-Cas9. They showed that combining MRTX849 with sarcoma Rous sarcoma viral oncogene homolog (SRC) inhibitors (like dasatinib, DGY-06-116, and bosutinib) synergistically enhances anti-tumor effects in patient-derived organoids. The study revealed that JUN upregulates ABCC1 to mediate resistance, while dasatinib inhibits the SRC-JUN pathway, boosting MRTX849 efficacy and overcoming resistance. Chen et al. [105] identified RUVBL1/2 as a potential target in YTHDF1-overexpressing colorectal cancer using CRISPR-Cas9. They confirmed that RUVBL1/2 knockout or inhibition with CB6644 and VNP-siRNA preferentially suppresses the growth of these cancer organoids, suggesting its potential as a drug target. These studies provide new targets, drug combination strategies, and approaches to overcoming drug resistance for cancer treatment (Summarized in Table 3) [[101], [102], [103], [104], [105]].

**Table 3:** Applications of clustered regularly interspaced short palindromic repeats (CRISPR) Screening in organoids studies.

Organoids origin	Organoids model	Types of CRISPR screening	Disease	Targets	Mechanisms	Drug validation	Clinical implications	Refs.
PDO	PDAC organoid	Reverse screening	Pancreatic cancer	ARID1A, BRCA2	Hypoxia response	Dasatinib, VE-821, emetine, ouabain	A high-fidelity *in vitro* model was provided for predicting and studying drug responses in pancreatic cancer, as well as evaluating individual drug susceptibility.	[101]
	Bladder cancer PDO	Forward screening	Bladder cancer	NPEPPS	Drug resistance	Tosedostat, cisplatin, Gemcitabine	Offering a new therapeutic strategy to overcome platinum drug resistance, which may improve the treatment outcome for bladder cancer patients and reduce treatment-related toxicity.	[102]
	Breast cancer PDO	Forward screening	Breast cancer	EGFR, CDK2, CDK13, GAK, PTK2, PRKDC	Synergistic mechanism of EGFR and FGFR1 inhibitors	Gefitinib, SR-4835, etc.	It provides new targets and therapeutic strategies for precision medicine, particularly for underrepresented African ancestry populations in clinical trials.	[103]
	NSCLC PDO	Forward screening	KRAS-G12C mutated NSCLC	ABCC1	Drug resistance	MRTX849, dasatinib	It provides important references for the development of new SRC inhibitors and ABCC1 inhibitors, which is helpful for promoting the research and development of related drugs and their clinical application.	[104]
	CRC PDO	Forward screening	Colorectal cancer	RUVBL1/2	MAPK/PI3K-Akt signaling pathway	CB6644, VNP-siRNA	Targeting RUVBL1/2 can inhibit YTHDF1-mediated oncogenic translation, providing a new strategy for precision treatment of colorectal cancer.	[105]
hPSCs	Human hepatocyte organoids	Forward screening	NAFLD	FADS2	Lipid metabolism	ACC, FAS, and DGAT2 inhibitors	It provides a HTS platform based on human organoids, which can be used to discover new therapeutic targets and drug screening, and contribute to the development of new therapies for NAFLD.	[106]
	Human hepatocyte organoids	Forward screening	DILI	BSEP	Bile acid transport	Indomethacin, Zileuton	A high-fidelity *in vitro* model is provided for predicting and studying the pathological mechanisms of DILI, as well as for evaluating individual susceptibility to drugs.	[107]
	Human brain organoid	Forward screening	TBI	KCNJ2, TDP-43	Calcium ion influx	ML133	Not only does it provide a new strategy for the treatment of TBI, but it also offers a reference for precision medicine in other neurodegenerative diseases.	[108]
	Human brain organoid	Forward screening	AD	Dkk1, PTEN	WNT signaling pathway, autophagy	Flibanserin, Ripasudil, Everolimus, etc.	By combining mathematical modeling with the pathological characteristics of human brain organoids, an efficient drug screening platform for AD has been developed, providing a new strategy for precision medicine.	[109]
	Human kidney organoids	Reverse screening	Renal tubular disease associated with KCNJ16 gene mutations	KCNJ16	Lipid accumulation and fibrosis	Simvastatin	It offers new insights into KCNJ16-related kidney diseases and support the development of personalized treatments.	[111]

PDO: patient-derived organoids; PDAC: pancreatic ductal adenocarcinoma; ARID1A: AT-rich interaction domain 1A; BRCA2: breast cancer 2; NPEPPS: puromycin-sensitive aminopeptidase; EGFR: epidermal growth factor receptor; FGFR1: fibroblast growth factor receptor 1; CDK2/13: cyclin dependent kinase 2/13; GAK: cyclin g-associated kinase; PTK2: protein tyrosine kinase 2; PRKDC: protein kinase, DNA-activated, catalytic subunit; KRAS: kirsten rat sarcoma viral oncogene homolog; NSCLC: non-small cell lung cancer; SRC: sarcoma viral oncogene homolog; ABCC1: ATP-binding cassette transporter C1; CRC: colorectal cancer; hPSCs: human pluripotent stem cells; MAPK: mitogen-activated protein kinase; PI3K: phosphatidylinositol 3-kinase; Akt: protein kinase B; NAFLD: non-alcoholic fatty liver disease; FADS2: fatty acid desaturase 2; ACC: acetyl-CoA carboxylase; FAS: fatty acid synthase; DGAT2: diacylglycerol O-acyltransferase 2; DILI: drug-induced liver injury; BSEP: bile salt export pump; TBI: traumatic brain injury; KCNJ2: potassium voltage-gated channel subfamily J member 2; TDP43: trans-activator regulatory (TAR) DNA-binding protein 43; AD: Alzheimer's disease; Dkk1: Dickkopf-1; PTEN: phosphatase and tensin homolog deleted on chromosome 10; KCNJ16: potassium voltage-gated channel subfamily J member 16.

### 5.2 CRISPR screening in hPSCs-derived organoids

Pioneering the field of organoids, Hendriks et al. [106] constructed a hepatocyte organoid model using CRISPR technology to simulate non-alcoholic fatty liver disease (NAFLD)-associated steatosis. They screened 17 candidate drugs and found that inhibiting fat synthesis (e.g., acetyl-CoA carboxylase (ACC), fatty acid synthase (FAS), and diacylglycerol O-acyltransferase 2 (DGAT2) inhibitors) and activating the farnesoid X receptor (FXR) pathway effectively reduced steatosis. Additionally, they developed the FatTracer platform, employing APOB^−/−^ and MTTP^−/−^ organoids for CRISPR knockout screening, and identified fatty acid desaturase 2 (FADS2) as a key regulator of steatosis. Its overexpression was shown to alleviate steatosis, providing new directions for NAFLD treatment. In addition, Shinozawa et al. [107] established a high-fidelity drug-induced liver injury (DILI) screening model using hPSCs-derived liver organoids. They validated the model's effectiveness by knocking out the bile salt export pump (BSEP) gene using CRISPR technology. Furthermore, they developed a high-throughput toxicity screening platform based on human liver organoids (LoT), which tested 238 marketed drugs (such as Indomethacin and Zileuton) for toxicity. The platform successfully predicted susceptibility to drug-induced cholestasis, providing a new tool for personalized drug toxicity research. Lai et al. [108] used CRISPRi in human brain organoids (BOs) to identify potassium inwardly rectifying channel subfamily J member 2 gene (KCNJ2) as a traumatic brain injury (TBI) therapeutic target. They simulated TBI with a high-precision ultrasound and found that the KCNJ2 inhibitor ML133 reduced neurodegeneration, especially in amyotrophic lateral sclerosis (ALS) and frontotemporal dementia (FTD) patient organoids with chromosome 9 open reading frame 72 (C9ORF72) mutations. Using induced pluripotent stem cells (iPSCs) derived from patient adult cells, Park et al. [109] used CRISPR-Cas9 to edit the ApoE ε4 allele and generate BOs. By combining mathematical models with a high-content screening system, they screened US FDA-approved drugs (such as Flibanserin, Ripasudil, and Everolimus) and identified compounds that could improve AD pathology. This work provides new strategies for precision medicine in AD treatment. Other human AD-BO models derived from human embryonic stem cells (hESCs) and induced using human blood serum are also available for further CRISPR screening studies [110]. Sendino Garví et al. [111] used CRISPR to create a KCNJ16-mutated kidney organoid model, showing lipid metabolism disorders and fibrosis. Drug screening revealed that simvastatin reduced lipid droplet accumulation and collagen deposition, offering a potential treatment strategy for KCNJ16-related kidney diseases. In the field of psychiatric disorders, BO-stress models have also been developed and shown to exhibit abnormalities in neurotransmitter secretion. This model can be combined with CRISPR screen to identify potential therapeutic targets for conditions such as anxiety and depression [112]. These studies demonstrate the significant potential of CRISPR technology combined with organoid models in advancing disease research and drug development (Summarized in Table 3) [[106], [107], [108], [109],^111^].

## 6 Challenges of CRISPR screening

### 6.1 Off-target effects

**Fig. 4:**
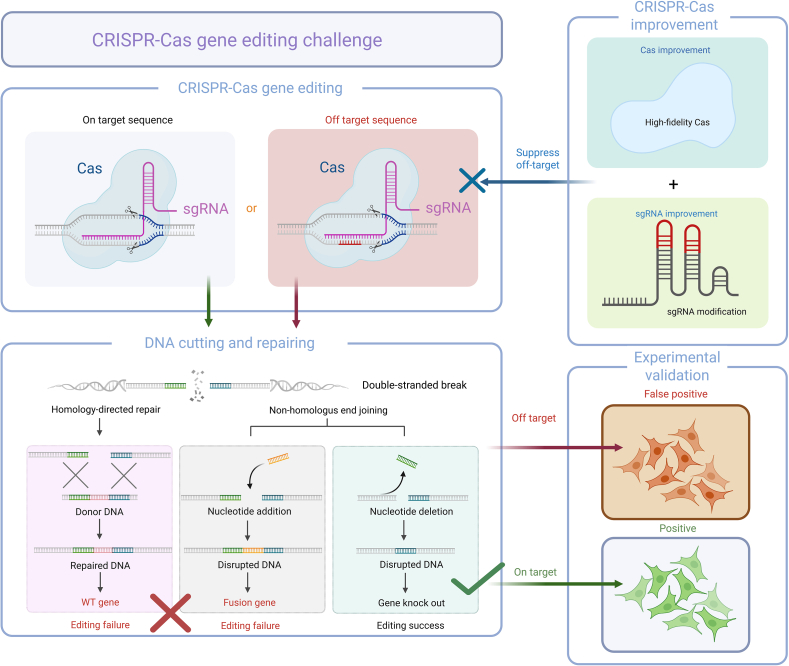
Schematic diagram of off-target effects. Clustered regularly interspaced short palindromic repeats (CRISPR) gene editing repairs double-strand breaks (DSBs) via homology directed repair (HDR) or non- homologous end joining (NHEJ). The stochastic nature of these repair mechanisms may lead to three outcomes: successful repair, fusion gene formation, or gene knockout. Even with on-target editing, the randomness of repair and low validation efficiency make isolating positive clones challenging. Unintended DNA breaks caused by off-target effect at non-target loci undergo similar stochastic repair, risking unintended knockouts or fusion proteins. These events generate false-positive clones and complicate data interpretation, potentially derailing screenings. Optimizing Cas9 and sgRNA design to minimize off-target activity is critical for improving CRISPR screening reliability.

Although CRISPR-Cas specificity is primarily determined by sgRNA-PAM recognition, tolerances such as 3–5 base pair mismatches between gDNA and sgRNA sequences [15], combined with structural flexibility in base pairing (e.g., wobble effects) [113], enables sgRNA binding to non-target DNA, driving off-target cleavage activity—a critical limitation for therapeutic applications. Minimizing off-target effects is crucial for the technology to be effective and accurate. Besides, the DSBs induced by CRISPR editing can cause chromosomal rearrangements, a harmful genomic reorganization where chromosomes shatter and rejoin randomly [114]. Although most cells cannot survive such changes, some may exhibit false positives due to the expression of fusion proteins or the induction of gene dysregulation [115] (Fig. 4).

Beyond off-target-induced gene function alterations, CRISPR screens are limited by mechanism-driven technical challenges: gene-independent effects of DSBs and chromosomal arm truncation-induced neighborhood effects. Cas9-induced DSBs may activate DNA damage response pathways (e.g., the p53 pathway), leading to loss of cellular fitness—an effect unrelated to the targeted gene's function but mainly associated with the copy number status of the targeted loci [116]. Subsequent studies have demonstrated that this is particularly pronounced in genomic regions with tandem duplications [117]. Furthermore, recent research has revealed that unintended Cas9-mediated whole chromosomal arm truncations may result in co-deletion of neighboring genes, generating a "gene neighborhood effect", where genes located on the same chromosomal arm may be erroneously classified as essential [118]. These limitations risk introducing artifacts or confounding effects in screening data, demanding rigorous validation in genomically unstable cancers where copy number variation (CNV) complexity exacerbates interpretation challenges.

Several CRISPR off-target detection methods have been developed, including the whole genome sequencing (WGS), QUIDE-seq [119], and DISCOVER-seq [120]. To reduce the off-target effect, two key strategies have been proposed: 1. Cas9 improvement: SpCas9, derived from *Streptococcus pyogenes*, is the most used Cas9 variant, though its specificity remains suboptimal and amenable to improvement. Researchers have developed several variants, such as eSpCas9 [121], SpCas9-HF1, hypaCas9, and EvoCas9, which include a proofreading mechanism that reduces the off-target binding by 90%, without affecting on-target activity [[122], [123], [124]]. Additionally, paired SpCas9 nickases, which cut only one DNA strand, minimize the off-target effect when two sgRNAs target adjacent strands [124]. Using Cas9 orthologs, such as SaCas9 from *Staphylococcus aureus*, which recognizes a rarer PAM sequence, also enhances the specificity [125]. 2. sgRNA improvement: Optimizing the sgRNA sequences is crucial for reducing off-target effects. Engineered sgRNA modifications, such as 5′-terminal guanine extensions (5′-GGX20) or truncation by 2–3 nucleotides, enhance CRISPR-Cas9 specificity by stabilizing guide-target interactions and reducing off-target binding [124,126]. In addition, chemical modifications, such as 2′-*O*-methyl-3′-phosphonoacetate (MP) incorporation or locked nucleic acids (LNA), further enhance the on-target precision by slowing Cas9 reactivity [127,128].

### 6.2 Complexity of data analysis

The effectiveness of CRISPR screening largely depends on the robustness and accuracy of data analysis, which transforms vast and complex raw sequencing data into biologically meaningful results. Currently, a variety of bioinformatics methods and computational tools are employed to process and interpret CRISPR screening data, enabling the detection of genes involved in cell survival, drug resistance, and disease progression. A key analytical step in CRISPR screening is relative abundance analysis, which quantifies changes in sgRNA or gene abundance between treatment and control groups. This method infers the impact of gene perturbation on phenotypes by calculating Log2 fold changes (LFC) after normalization [129]. Common normalization strategies include RPKM/CPM [130], which adjusts read counts by sequencing depth, and Loess regression, which corrects biases associated with guanine-cytosine (GC) content and sequence characteristics. Via this approach, genes essential for cell viability or function can be systematically identified.

To statistically assess gene essentiality in CRISPR screening, computational tools are broadly categorized into supervised and unsupervised methods. Supervised tools such as BAGEL2 [131] utilize predefined reference gene sets—typically known essential and non-essential genes—to train Bayesian models, making them particularly effective for targeted or small-scale screens where prior biological knowledge is available. However, their reliance on high-quality reference datasets limits their applicability in poorly characterized systems or non-genome-wide screens, where such references may be incomplete or biased [132]. In contrast, unsupervised methods like MAGeCK [133] operate independently of reference genes, employing a negative binomial distribution to model sgRNA abundance changes across the genome. These tools are well-suited for hypothesis-free, genome-wide studies but are more sensitive to noise and batch effects, which can obscure true biological signals in limited datasets. Beyond statistical modeling, CRISPR screening in cancer models faces biological confounders such as CNVs and DSB toxicity, which may introduce false-positive or false-negative results [134,135]. To address these issues, tools like CRISPRcleanR [136,137] and BAGEL2 [131] correct for CNV-linked bias, while Chronos [134,138] and MAGeCK [133] adjust for guide efficiency and chromosomal arm effects. CRISPRcleanR and arm-corrected Chronos are the only methods able to correct both type of biases. CRISPRcleanR provides enhanced versatility by enabling CNV correction without prior genomic input, and can operate effectively on individual screens.

Quality control and reproducibility are critical for ensuring reliable CRISPR screening outcomes. Targeted metric screening frameworks have been introduced to monitor data integrity and ease post-screening analysis. For instance, Behan et al. [35] developed a tumor dependency resource based on 324 human cancer cell lines, enhancing target prioritization and translational value. Similarly, Iannuzzi et al. [139] created an R package with a reference dataset to assess screening quality in HT-29 colon cancer cells, enabling benchmarking and standardized reporting. However, reproducibility can still be affected by subjective data interpretation and analytical variability. To address this, bioinformatics tools are increasingly designed to automate workflows, reduce human error, and improve accuracy. Programming languages such as R and Python remain essential in building these pipelines, supporting data normalization, statistical analysis, visualization, and quality assessment in CRISPR screening studies.

**Fig. 5:**
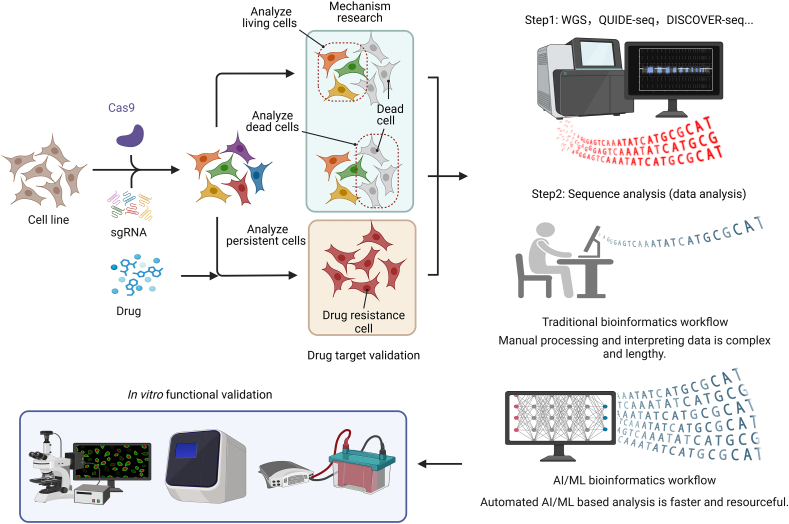
Clustered regularly interspaced short palindromic repeats (CRISPR) screening and data analysis. High-throughput CRISPR screening faces challenges: (1) Genome-wide single guide RNAs (sgRNAs) library design generates massive sequencing data, with off-target effects amplifying complexity (2) Screening models (e.g., mechanistic studies vs. drug discovery) dictate analytical workflows. Human errors during sample preparation may introduce biases. The integration of artificial intelligence (AI)/machine learning (ML) with CRISPR technology streamlines data analysis and enhances precision.

With the increasing scale and complexity of CRISPR datasets, artificial intelligence (AI) and machine learning (ML) have emerged as essential tools for enhancing data analysis (Fig. 5). AI models like DeepCRISPR [140] integrate sequence and epigenetic features to predict on-target efficiency and off-target risks, improving sgRNA design and reducing experimental noise. TIGER [141], another AI-driven tool, incorporates chromatin accessibility and sequence homology into sgRNA selection, offering context-aware guide design that enhances screening precision, especially in repetitive or mutation-rich genomic regions. Beyond off-target prediction, AI also aids in systematic CRISPR system discovery. The CHOOSER framework, for instance, applies protein foundation models to identify novel CRISPR-Cas systems with self-processing capabilities, discovering thousands of previously unrecognized candidates [142]. Furthermore, The ML models can predict gene function specificity, helping to reduce library size and experimental redundancy. One such model identified a set of 100–300 informative genes sufficient to predict loss-of-function effects across over 18,000 genes, demonstrating how ML can increase screening efficiency while retaining predictive power [143].

Despite these advancements, CRISPR screening data analysis continues to face substantial challenges. These include data heterogeneity, large-scale sequencing noise, and limitations of current analytical algorithms. Analysts must contend with issues such as variable library complexity, differences in sequencing depth, and inconsistent polymerase chain reaction (PCR) amplification—all of which can distort sgRNA abundance measurements. Moreover, connecting gene perturbations to phenotypic outcomes remains complex, especially when gene effects are subtle or context-dependent. In summary, while CRISPR screening offers unprecedented potential for functional genomics and drug target discovery, its success depends heavily on the quality and precision of data analysis. Addressing the computational challenges—ranging from off-target noise and CNV bias to reference dependence and AI integration—will be essential for extracting biologically actionable insights. Continued innovation in bioinformatics, particularly through AI-enhanced tools and quality control frameworks, will drive the reliability, scalability, and translational impact of CRISPR-based research.

### 6.3 Model limitations

The CRISPR-Cas system holds great promise; however, its application in real-world scenarios is limited by significant challenges across various cell and animal models. Firstly, the challenge of delivery stands as a primary obstacle. The efficacy and accuracy of CRISPR-Cas-mediated gene editing are contingent upon the intracellular expression and longevity of the editing components. While the precise delivery of Cas/sgRNA to target cells or specific animal tissues is crucial for successful gene editing, achieving this in practical research settings is often a complex task, potentially resulting in inadequate or failed editing results [144]. Secondly, we encounter limitations in functional gene studies. It is common to face homozygous lethality with knockouts, which implies that screening efforts can only retrieve heterozygous clones. This limitation may compromise the effectiveness of subsequent functional validation studies. Lastly, the inescapable off-target effects can spawn false positives, further complicating the drug target validation process and casting a veil of uncertainty over the outcomes [145]. These challenges underscore the need for innovative solutions to enhance the delivery, specificity, and reliability of CRISPR/Cas applications in clinical and research contexts.

### 6.4 Ethical and regulatory issues

While CRISPR-based show promise in treating various diseases, significant ethical concerns persist, particularly regarding off-target editing and irresponsible clinical practices. As highlighted in numerous studies, off-target effects lead to severe complications, such as loss-of-function mutations or mis-repair of disease-related genes due to incorrect DNA binding and cleavage outside the targeted sequence genetic changes, including chromosomal rearrangements, underscore the necessity of thorough off-target assessment before advancing CRISPR-Cas9 for human genome editing. Despite the clear understanding of CRISPR's mechanism, the off-target effects, which may impact edited organisms and their descendants, remain poorly controlled.

The other issue concerning with CRISPR-Cas is its potential for unlawful or unethical use in human experimentation. In November 2018, Scientist Jiankui He claimed to have used CRISPR-Cas9 to create gene-edited infants resistant to HIV [146], a revelation that sparked widespread condemnation across global academic and research communities. This event indicates the critical need for strict regulations prohibiting the use of genome editing in human embryos until targeting precision and safety can be guaranteed.

Moreover, in the process of combining CRISPR screening technology with drug screening, ethical issues are also an important aspect that cannot be ignored. During drug screening, it is essential to strictly assess and control the off-target effects of CRISPR, using advanced technologies (such as AI-assisted methods) to reduce off-target risks and validate the specificity and accuracy of drug screening results through multiple approaches in experiments. It helps to minimize the risks of abnormal cell function, oncogenesis, and other potential hazards. When drug screening involves cells directly sourced from patients or clinical trials, obtaining informed consent from the subjects is of vital importance. Additionally, the large amounts of genomic data generated during CRISPR-based drug screening, which contain sensitive personal information of patients, must be strictly managed and controlled to ensure the security of all data. Therefore, it is essential to adhere to ethical guidelines, respect human dignity, and ensure that any clinical applications of CRISPR comply with established laws and regulations.

## 7 Perspectives on CRISPR screening in drug and target discovery

### 7.1 Improving off-target control

To mitigate off-target effects, future strategies may focus on several key areas. Firstly, optimizing delivery methods can enhance targeting efficiency and reduce off-target impacts. For instance, ribonucleoprotein (RNP) delivery, where the CRISPR-Cas system is not integrated into the host genome, offers a short lifespan for RNA/protein, limiting off-target risks. Secondly, further engineering of Cas enzymes with higher affinity and specificity improves precision in target site binding and DNA cleavage. Thirdly, designing longer or more reliable gRNAs could enhance target site binding accuracy. Lastly, linking Cas enzymes with proofreading enzymes could correct off-target edits, providing a rapid solution to mitigate unintended genome modifications. These approaches are critical directions for improving the CRISPR-Cas technology. A recent study equipped ML to train BE-Hive and achieved efficient base editing genotype and lower off-target impact in a target library analysis that included 38,538 genomically integrated targets [147]. Another study employed CRISPR guide RNA assisted reduction of damage (CRISPR GUARD), which uses short-guide RNAs to compete with on-target gRNA, protecting off-target sites. This method effectively reduces off-target mutations while maintaining on-target editing efficiency with both the Cas and base editors, offering potential for eliminating off-target effects in future CRISPR applications [148].

### 7.2 Integrative multiple screening strategies

Combining CRISPR screening with RNAi and compound screening enhances drug development by providing comprehensive understanding of gene function and drug mechanisms, thus expanding pharmaceutical innovation. Conducting CRISPR and RNAi screens concurrently allows to cross-validate their findings, ensuring that the results are robust and not merely byproducts of a single technological approach [149]. Given the inherent potential for off-target effects in both RNAi and CRISPR technologies, the parallel application of these screens serves as a dual-validation strategy, significantly minimizing the incidence of false positives. By leveraging CRISPR and RNAi to create cell models with tailored genetic profiles, followed by compound screening within these models, enables uncover novel therapeutic targets, elucidate the intricacies of drug action, and devise more potent treatment regimens. This integrated methodology not only expedites the drug development pipeline but also enhances the likelihood of successful new drug introductions to the market.

### 7.3 Clinical translation

The journey of translating CRISPR technology from the confines of the laboratory to the forefront of clinical practice is intricate and involves a concerted, multidisciplinary approach. It demands the collective expertise of biologists, medical professionals, ethicists, legal specialists, and regulatory bodies to ensure the technology's safety, efficacy, and compliance with standards. Firstly, CRISPR’s direct clinical applications are already making an impact, with the technology being used to correct genetic mutations in patients, such as hereditary blindness [150], and to enhance immune cells like CAR-T cells for cancer treatment [151]. The foundation for these direct applications lies in a deep understanding of the disease mechanisms, which have undergone rigorous functional validation. Secondly, CRISPR screening is instrumental in uncovering critical disease targets. For many diseases with obscure pathogeneses, CRISPR technology serves as a powerful tool to identify key functional genes, thereby providing potential therapeutic targets. Lastly, CRISPR technology plays a pivotal role in preclinical research for drug target validation. HTS of small molecules based on specific models yields a plethora of active compounds with unknown targets. Elucidating the mechanisms of action of these drugs is crucial before they can be considered for clinical application. CRISPR screening for drug target validation not only uncovers the targets of these drugs but also clarifies their mechanisms, thereby hastening their transition to clinical use.

## 8 Conclusion

Over the past decade, CRISPR screening has evolved from an emerging technology to a powerful tool in drug discovery, revolutionizing the identification of drug targets, understanding drug resistance, and accelerating drug development. This advancement is attributed to the refinement of sequencing techniques and the creation of comprehensive whole-genome sgRNA libraries, enhanced by synthetic biology, organoid technology and AI-driven molecular engineering. CRISPR screening enables precise, large-scale genome editing, improving the identification of genes and pathways implicated in diseases. Additionally, when combined with drug screening, CRISPR helps pinpoint drug-target interactions and aids in reverse identification of drug targets through drug-resistant clones. The integration with organoid technology has also greatly enhanced the efficiency of CRISPR in target discovery and development. Despite its great potential, challenges such as off-target effects, delivery limitations, and data analysis complexity remain. Ongoing improvements in sgRNA design, Cas9 variants, and delivery systems are essential for refining the technology. Moving forward, CRISPR screening is expected to remain central to drug research and development, offering transformative potential in discovering novel therapeutic targets and advancing precision medicine.

Overall, CRISPR screening technology holds immense potential to revolutionize drug development by enabling precise identification of novel drug targets and therapeutic mechanisms, offering more effective and safer treatments for complex diseases like cancer and genetic disorders. Its integration with organoid technology, AI and big data promises to enhance the efficiency and accuracy of the entire drug discovery process, fostering greater collaboration between academia and industry. This technological advancement will reshape drug development, driving innovation and significantly improving patient outcomes.

## CRediT authorship contribution statement

**Yao He:** Writing – review & editing, Writing – original draft. **Xiao Tu:** Writing – review & editing, Writing – original draft. **Yuxin Xue:** Writing – review & editing, Writing – original draft. **Yuxuan Chen:** Writing – review & editing, Conceptualization. **Bengui Ye:** Writing – review & editing. **Xiaojie Li:** Writing – review & editing. **Dapeng Li:** Writing – review & editing, Writing – original draft, Funding acquisition, Conceptualization. **Zhihui Zhong:** Writing – review & editing, Writing – original draft, Funding acquisition, Conceptualization. **Qixing Zhong:** Writing – review & editing, Writing – original draft, Funding acquisition, Conceptualization.

## Declaration of competing interest

The authors declare that they have no known competing financial interests or personal relationships that could have appeared to influence the work reported in this paper.
